# Surgery to treat autonomic dysreflexia due to cubital tunnel syndrome in cervical spinal cord injury: a case report

**DOI:** 10.1038/s41394-026-00750-4

**Published:** 2026-10-14

**Authors:** Kevin He, Brian Chin, Jonah P. Orr, Lucas Rohrer, Ida K. Fox

**Affiliations:** Division of Plastic and Reconstructive Surgery, Washington University in St. Louis, St. Louis, Missouri USA

**Keywords:** Spinal cord diseases, Pathology

## Abstract

**Introduction:**

Autonomic dysreflexia (AD) is a potentially fatal syndrome in people with spinal cord injury (SCI) marked by episodic hypertension with flushing, diaphoresis, and bradycardia. Common etiologies include bladder distension and bowel impaction. Compressive neuropathies lack attention as a potential precipitant of AD. This report describes an individual with cervical SCI and with recurrent AD treated with ulnar nerve decompression surgery.

**Case Presentation:**

A 74-year-old woman with a remote history of C6 SCI (American Spinal Injury Association Impairment Scale Grade B) presented to clinic with recurrent episodes of AD that had been refractory to standard treatment measures. History and clinical examination were suggestive of ulnar nerve compressive neuropathy at the elbow - cubital tunnel syndrome. The diagnosis was confirmed with electrodiagnostic studies and neuromuscular ultrasound. Treatment with surgery successfully resolved her episodes of AD.

**Discussion:**

This case identifies compressive neuropathy as a treatable cause of AD in individuals with cervical SCI. When more common etiologies of AD are excluded, clinicians should consider compressive peripheral neuropathies as potential causes of AD. Surgical decompression may alleviate symptoms of AD in select cases. Further research should investigate the prevalence and optimal management of compressive neuropathy in people with SCI.

## Introduction

**Fig. 1:**
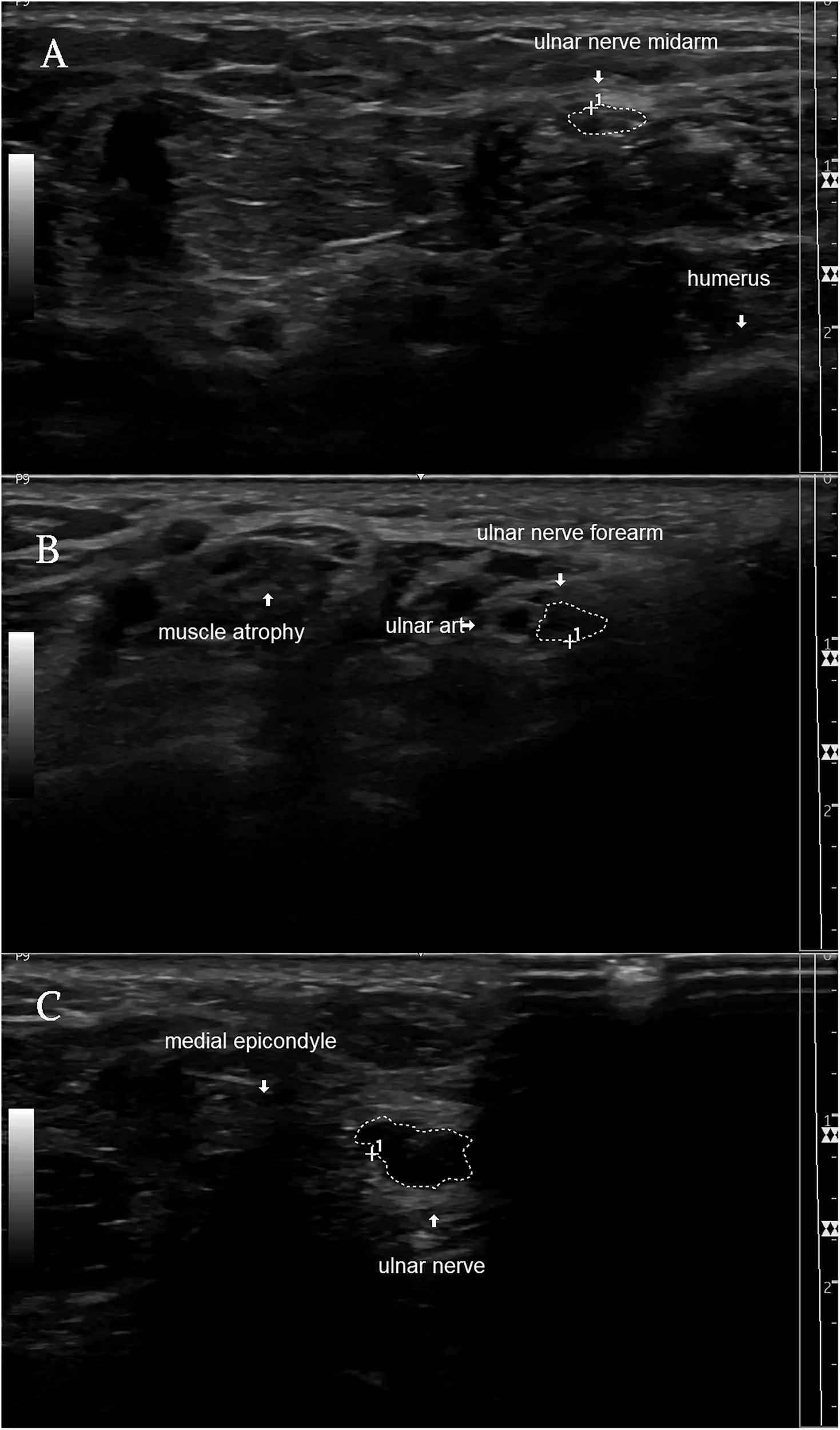
Neuromuscular Ultrasound of the Affected Ulnar Nerve. Neuromuscular ultrasound of the left ulnar nerve demonstrated normal cross-sectional area at the (**A**) mid-arm (5.9 mm^2^) and (**B**) forearm (6.8 mm^2^), with marked focal enlargement at the (**C**) elbow (25.0 mm^2^), consistent with ulnar nerve compression at the cubital tunnel.

Autonomic dysreflexia (AD) is potentially life-threatening to people with SCI and presents with hypertension, flushing, diaphoresis, and headache. SCI predisposes to AD by disconnecting supraspinal autonomic control centers from sympathetic neurons below the level of injury, preventing appropriate inhibitory regulation of sympathetic outflow. With this loss of supraspinal inhibition, noxious stimuli below the level of SCI precipitate unopposed sympathetic response, leading to symptoms characteristic of AD. Compensatory vagal responses above the lesion are often insufficient to counteract this hypertensive crisis and may lead to the bradycardia associated with AD [1]. (Fig. 1).

The frequency, severity, and potential causes of AD depend on the level and completeness of the SCI [2]. Complete and higher-level SCI are associated with greater risk, frequency, and severity of AD [2, 3]. For SCI at or above the T6 level, urologic, gastrointestinal, visceral, and cutaneous insults are well recognized as precipitants of AD [1, 4]. Since noxious stimuli must occur below the level of the SCI to precipitate AD [1], more cephalad levels of SCI, are potentially susceptible to other etiologies of AD such as upper extremity compressive neuropathies. Upper extremity compressive neuropathies are highly prevalent in people with SCI ranging from 40–73% [5], with highest occurrence in individuals with higher-level SCI injuries [3] and manual wheelchair users [6–8]. Although compressive neuropathies are common after SCI, their role as potential triggers of AD remains largely unexplored in the literature. In this case report, compressive neuropathy produced sufficient nociceptive input to trigger episodic AD in a person with cervical SCI.

## Case presentation

### History and presentation

A 74-year-old female was referred to our Upper Extremity and Peripheral Nerve clinic for evaluation of AD. She sustained a C6 ASIA/ISCoS International Standards for Neurological Classification of Spinal Cord Injury (ISNCSCI) Impairment Scale Grade B SCI in a motor vehicle collision 50 years ago. She had undergone left side tendon transfers to restore pinch and grip at 3 years post injury (pronator teres to flexor digitorum profundus and brachioradialis to flexor pollicis longus).

For five years prior to presentation, she experienced symptoms concerning for AD, particularly with activities requiring repeated elbow flexion and extension such as propelling her wheelchair, sewing, or driving. She has used her right hand to steer and her left hand to grip the acceleration control. She endorsed worsening symptoms when using her left hand to control the speed of her vehicle; this was also associated with elbow pain. She measured and noted her blood pressure was elevated to a systolic pressure of up to 190 mmHg from a baseline of 110 mmHg. Although her symptoms resolved with rest, they limited her driving, sewing and overall function and independence.

A comprehensive evaluation completed by a multidisciplinary SCI clinic did not identify other common causes of her AD episodes. This individual followed a routine of intermittent self-catheterization and daily stool evacuation. She had a history of stable coxalgia which was managed with hydrocolloid dressings and pressure distributing cushions. She did not have contracture of the left elbow. Preoperative radiographic studies demonstrated moderate degenerative changes consistent with osteoarthritis of the elbow.

Exam showed preserved strength at her shoulder and elbow, indicating injury pattern consistent with C7 AIS B. She was able to activate left hand closure using her tendon transfers and tenodesis. There was focal sensitivity of the left ulnar nerve with palpation. She endorsed feeling diaphoretic when the ulnar nerve was manually compressed and the left elbow held in flexion. These clinical findings were suggestive of cubital tunnel syndrome.

#### Investigations

Nerve conduction studies (NCS) showed a severe non localizable left ulnar neuropathy. There was no response with proximal stimulation of the ulnar nerve (non-recordable ulnar motor NCS), recorded at the left abductor digiti minimi (ADM) and first dorsal interossei (FDI). Stimulation of the ulnar nerve at the level of the wrist showed diminished compound motor action potential (CMAP) amplitudes and prolonged latencies. The sensory ulnar NCS was limited by technical issues. By contrast, the left median motor NCS showed small CMAP but normal latency and conduction velocity. The median sensory NCS were recordable with low amplitude but normal latency and velocity.

Electromyography (EMG) showed decreased insertional activity of the FDI with +1 positive sharp waves and absent spontaneous activity of the flexor carpi ulnaris (FCU). There were no motor unit action potentials in either the FDI or FCU. (Table 1)

**Table 1:** Nerve Conduction Studies (NCS) and Electromyography (EMG) Results.

Electrodiagnostic Results						
Motor nerve conduction studies						
Nerve Site	Amplitude (mV)	Latency (ms)	Segments	Distance (mm)	Latency Difference (ms)	Velocity (m/s)
L Median-APB						
Wrist	2.4	3.33	Wrist-APB	70	–	–
Elbow	2.2	8.4	Elbow–wrist	255	5.06	50.4
L Ulnar-ADM						
Wrist	0.3	6.46	Wrist-ADM	70	–	–
Below elbow	NR	NR	Below elbow-wrist	–	NR	–
L Ulnar-FDI						
Wrist	0.5	4.71	Wrist-FDI	–	–	–
Below elbow	NR	NR	Below elbow-wrist	–	–	NR
Sensory nerve conduction studies						
Nerve / Site	Amplitude (µV)	Onset Latency (ms)		Peak Latency (ms)	Distance (mm)	Velocity (m/s)
L Median digit 2	7	2.7		3.2	140	52
L Ulnar digit 5	0	0		0	140	0

Electromyography (EMG)										
Muscle	Insertional Activity	Spontaneous			Motor unit action potentials					
		Fibrillation	Positive Sharp Wave	Fasciculation	Duration	Amplitude	Polyphasia	Recruitment	Activation	Comment
L Deltoid	Normal	None	None	None	Normal	Normal	None	Normal	Normal	
L Triceps brachii (lat. head)	Normal	None	None	None	Moderately increased	Slight increase	None	Moderately Reduced	Normal	Chronic C7 radiculopathy
L Biceps brachii	Normal	None	None	None	Normal	Normal	None	Normal	Normal	
L Extensor Digitorum Communis	Normal	None	None	None				None	None	
L Flexor Carpi Radialis	Normal	None	None	None	Slight increase	Normal	None	Normal	Normal	
L First dorsal interosseous	Decreased	None	1+	None				None	None	
L Abductor Pollicis Brevis	Slight increase	None	None	None				None	None	
L Flexor Carpi Ulnaris	Absent	None	None	None				None	–	

Summary of preoperative electrodiagnostic findings demonstrating severe left ulnar neuropathy.

A neuromuscular ultrasound of the left ulnar nerve demonstrated normal diameter of the ulnar nerve at the mid-arm (5.9mm^2^, Fig. 1A) and forearm (6.8mm^2^, Fig. 1B) with focal enlargement at the elbow (25.0mm^2^, Fig. 1C).

#### Management

Given these findings, compressive neuropathy of the ulnar nerve at the elbow or cubital tunnel syndrome, was considered as a potential etiology for the episodes of AD. Intensive therapy, which included nerve glides, nighttime splinting and activity modification; was completed. Having exhausted conservative strategies, operative treatment was discussed. After reviewing the potential benefits and risks of surgery, she decided to proceed with surgery.

**Fig. 2:**
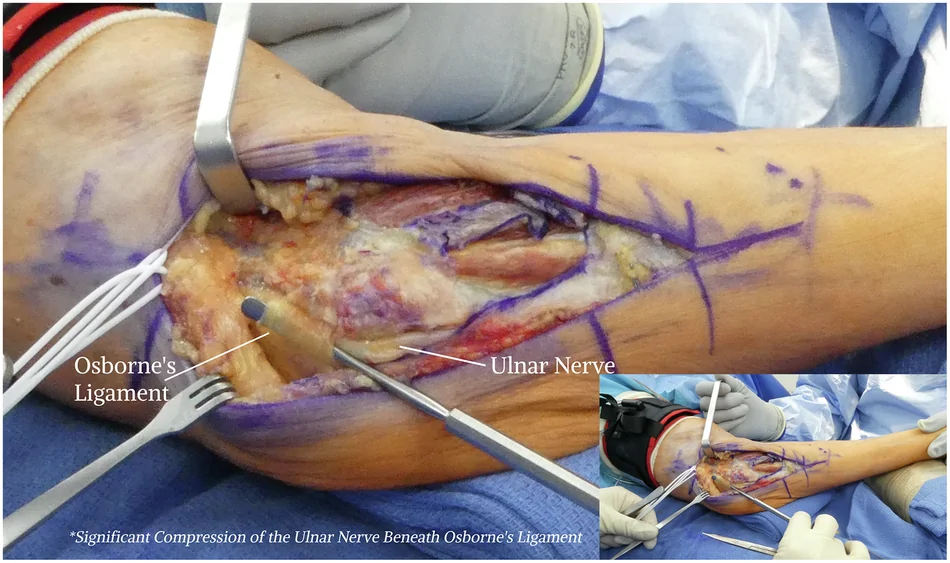
Intraoperative Photo Demonstrating Compression of the Ulnar Nerve. Intraoperative photograph demonstrating substantial compression of the left ulnar nerve beneath Osborne’s ligament during cubital tunnel decompression.

**Fig. 3:**
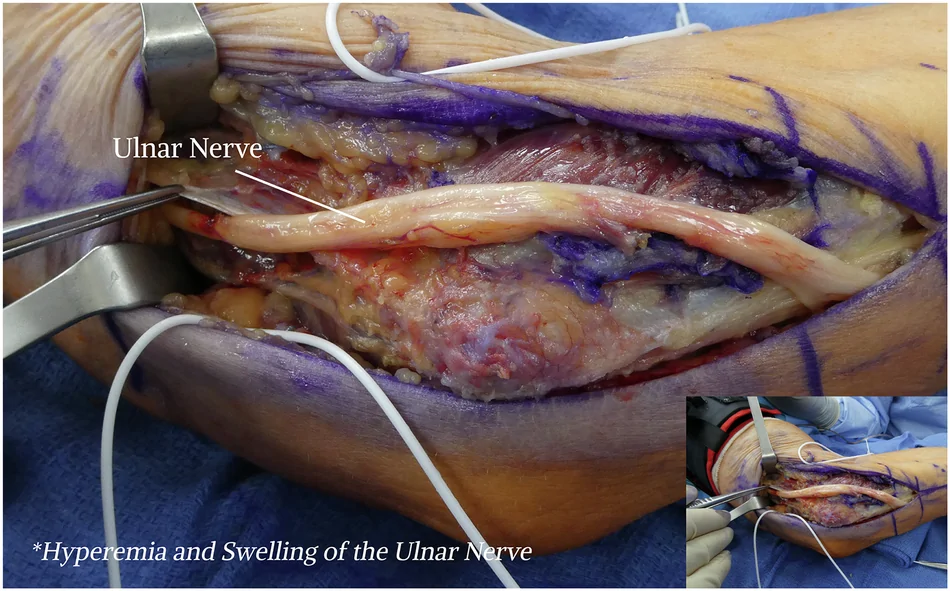
Intraoperative Photo Demonstrating Edema of the Ulnar Nerve. Intraoperative photograph of the left ulnar nerve demonstrating focal hyperemia and edema at the site of compression.

She underwent an uncomplicated ulnar nerve decompression with anterior submuscular transposition at the elbow under general anesthesia. Intraoperative findings demonstrated substantial compression of the ulnar nerve beneath Osborne’s ligament (Fig. 2) with focal hyperemia and edema (Fig. 3). Intraoperative electrical stimulation of the ulnar nerve produced tachycardia and hypertension and as well as muscular contraction of the ulnar innervated flexor digitorum profundus and intrinsic muscles. These findings were consistent with intact C8-T1 lower motor neurons below the level of SCI in keeping with the known pathophysiology of AD.

#### Outcome

After surgery, the operative extremity was splinted, and activity was limited to non-weight bearing for a month. During this period, she required substantial additional caregiver support for transfers and ADLs. She experienced worsening of her coxalgia, which was managed by updated seat pressure mapping and modification of her wheelchair and vehicle seat pads. At her 5-month postoperative visit, she reported complete resolution of her AD with driving and other activities requiring left elbow flexion such as sewing and gardening.

## Discussion

Compressive neuropathies are an underrecognized as an etiology for AD in individuals with cervical SCI. In this report, severe cubital tunnel syndrome was identified as the precipitant for recurrent, unexplained AD. Clinical suspicion was confirmed through comprehensive evaluation including 1) the history of precipitating activities, 2) physical exam findings notable for reproduction of AD symptoms with external ulnar nerve compression and elbow flexion, and 3) supporting EMG/NCS and neuromuscular ultrasound findings. Intraoperative findings showing electrical stimulation that produced ulnar innervated muscle contracture coupled with a lack of volitional control of these muscles further supported the presumptive diagnosis. The resolution of AD following nerve decompression and transposition surgery demonstrates that peripheral compression can lead to AD in SCI and should be in the differential diagnosis when more common causes are excluded.

Several reports have documented treatable neuropathic sources of AD in individuals with SCI. Structural spinal pathology is a well described source of noxious stimuli, with several case reports describing posture-induced AD associated with Charcot spine resolving after surgical correction [9–12]. Similarly, hardware failure [13], spinal pseudoarthrosis [13], and cervical stenosis [14] have also been identified as treatable precipitants of AD. These examples establish that sustained nociceptive input from central/spinal neuropathic sources are a recognized and treatable etiologies for provoking AD.

In contrast, peripheral compressive neuropathies are largely unexplored as neuropathic causes of AD. Despite the fact that compressive neuropathies are highly prevalent in SCI [8, 15], they are often underdiagnosed [16–18] and undertreated [19] in this population. When identified in SCI, conservative [20], glucocorticoid injection [21], or surgical management [22, 23] may be effective and safe treatment options. For instance, case reports describe resolution of compressive radial neuropathy in C6 AIS A SCI with mobility exercises and vitamin B supplementation [20]. In another case report, suprascapular nerve compression in T9 AIS C SCI resolved with glucocorticoid injection [21]. Other case reports describe successful surgical decompression in treating shoulder pain arising from radial and axillary nerve compression (C6 AIS A and T4 SCI) [22] and anterior interosseous nerve neuropathy (C7 AIS B) [23]. Additional studies report successful median [5, 16, 24] and ulnar nerve decompression in individuals with SCI [5].

Although decompression can be effective [24], the decision to pursue surgery requires careful weighing of functional tradeoffs. For an individual with SCI, the requisite postoperative non-weight bearing leads to substantial, temporary loss of independence [25]. In this case, the intervention was pursued only after a thorough diagnostic workup identified cubital tunnel as the AD trigger and conservative measures had been exhausted. Postoperative recovery led to the expected short-term functional decline. Complete resolution of AD symptoms with resumptions of driving and other elbow flexion activities required extensive therapy and took five months to achieve (treatment of perioperative worsened coxalgia also contributed to the length of time required).

One additional complicating factor is the individual’s history of prior tendon transfers on the affected limb. Although an established method for functional restoration in SCI [26], tendon transfers can alter local anatomy and create a risk for secondary nerve compression. Case reports (in people without SCI) describe ulnar compression with and without atypical neurologic symptoms following tendon transfers [17, 27] which resolved with rerouting and neurolysis [17, 18, 27]. In SCI, Oiknine et al. described ulnar neuropathy after a biceps-to-triceps tendon transfer that was managed with decompression and anterior transposition at the elbow [28].

The findings from this single case observation are subject to several limitations. Other factors such as patient comorbidities [5], severity of SCI [29, 30], subjective pain thresholds [31], and potential subclinical noxious inputs [32, 33] often make it difficult to attribute AD to a single etiology. Furthermore, while the temporal association between surgery and symptom resolution is compelling, it does not establish causation, and further long-term follow-up will also be needed to confirm the durability of this treatment.

## Conclusion

This case suggests that peripheral compressive neuropathy may serve as a sufficient, correctable trigger for AD. Given the high prevalence and diagnostic challenges, clinicians should maintain a high index of suspicion for compressive neuropathies, especially when more common etiologies of AD are absent. Timely identification and treatment can resolve AD episodes, restore associated loss of function, and improve quality of life.

## Data Availability

The datasets generated during and/or analysed during the current study are available from the corresponding author on reasonable request.
